# Genome assembly of the temporary socially parasitic spiny ant *Polyrhachis lamellidens* and its host *Camponotus japonicus*

**DOI:** 10.1093/dnares/dsaf005

**Published:** 2025-03-01

**Authors:** Hironori Iwai, Yu Kurihara, Nobuaki Kono, Masaru K Hojo, Katsushi Yamaguchi, Shuji Shigenobu, Mamiko Ozaki, Akiko Koto, Kazuharu Arakawa

**Affiliations:** Cellular and Molecular Biotechnology Research Institute, National Institute of Advanced Industrial Science and Technology, Ibaraki 305-8566, Japan; Institute for Advanced Biosciences, Keio University, Yamagata 997-0017, Japan; Postdoctoral Research Fellowships, Japan Society for the Promotion of Science, Tokyo 102-0083, Japan; Graduate School of Media and Governance, Keio University, Kanagawa 252-0882, Japan; Institute for Advanced Biosciences, Keio University, Yamagata 997-0017, Japan; Graduate School of Media and Governance, Keio University, Kanagawa 252-0882, Japan; Faculty of Environment and Information Studies, Keio University, Kanagawa 252-0882, Japan; School of Biological and Environmental Sciences, Kwansei Gakuin University, Hyogo 669-1330, Japan; Trans-Scale Biology Center, National Institute for Basic Biology, Okazaki 444-8585Japan; Trans-Scale Biology Center, National Institute for Basic Biology, Okazaki 444-8585Japan; Life Science Center for Survival Dynamics, Tsukuba Advanced Research Alliance (TARA), University of Tsukuba, Ibaraki 305-8577, Japan; Graduate School of Science, Kobe University, Kobe 657-8501, Japan; KYOUSEI Science Center for Life and Nature, Nara Women’s University, Nara 630-8263, Japan; Graduate School of Engineering, Kobe University, Kobe 657-8501, Japan; RIKEN Center for Biosystems Dynamics Research, Kobe 650-0047, Japan; Cellular and Molecular Biotechnology Research Institute, National Institute of Advanced Industrial Science and Technology, Ibaraki 305-8566, Japan; Computational Bio Big Data Open Innovation Laboratory, National Institute of Advanced Industrial Science and Technology, Ibaraki 305-8566, Japan; Institute for Advanced Biosciences, Keio University, Yamagata 997-0017, Japan; Graduate School of Media and Governance, Keio University, Kanagawa 252-0882, Japan; Faculty of Environment and Information Studies, Keio University, Kanagawa 252-0882, Japan

**Keywords:** genome assembly, social insect, ant, social parasite

## Abstract

*Polyrhachis lamellidens* is a temporary socially parasitic ant. The newly mated *P. lamellidens* queen takes over a colony of several *Camponotus* ant species and uses the labour of the host workers in the early stages of social parasitism. To facilitate genomic resources for these species, we assembled and annotated the chromosomal genome of *P. lamellidens* using the 10× Genomics linked-read and Hi-C sequencing, and the draft genome of its host, *Camponotus japonicus*, using long-read sequencing with the Revio system. The *P. lamellidens* chromosomal genome assembly is 214.1 Mb, 95.5% BUSCO completeness, and contains 13,703 protein-coding genes. The *C. japonicus* draft genome assembly is 314.2 Mb, 99.0% BUSCO completeness, and contains 11,207 protein-coding genes. Genome-wide phylogeny and synteny analysis confirmed the phylogenetic position of *P. lamellidens* and *C. japonicus*, and a high level of synteny with the genome of both ant species. In addition, *P. lamellidens* possesses nearly identical chemosensory proteins to its host, *C. japonicus*, and these genes tended to exhibit higher expression levels in the newly mated queen. The genome assemblies of *P. lamellidens* and its host *C. japonicus* provide a valuable resource for the molecular biological and bioinformatic basis for studying the strategy of social parasitism in ants.

## Introduction

Ants are one of the most prosperous groups of eusocial insects. In general, ants establish and maintain colonies without relying on other ant species. However, some species depend for part or all of their lives on societies formed by other species. This strategy is called social parasitism and occurs in eusocial insects, especially ants, wasps, and bees.^1–4^ In ants, a variety of social parasitism strategies (e.g. inquilinism, dulosis, and temporary social parasitism) have convergently evolved in different taxa.^1,3,4^

With the recent development of sequencing technology, omics studies have been conducted in various ant species,^5,6^ starting with *Camponotus floridanus* and *Harpegnathos saltator*.^7^ Omics-level studies have also been conducted in socially parasitic ant species and are beginning to explore at the molecular level the evolutionary background from which the strategy of social parasitism evolved. However, many of these omics studies have focused on inquilines (parasites lacking the worker caste responsible for labour and rely on host workers for all labour) or dulotic ants (parasites with the worker caste, but rely on host workers for all labour) and their hosts,^8–15^ and there are still few examples of studies focusing on temporary socially parasitic ants (parasites temporarily use the labour of the host workers in the early stages of colony foundation). In addition, most of the socially parasitic ant species mentioned above follow Emery’s rule that socially parasitic ants and their hosts tend to be closely related species,^16^ and the genomes of species that deviate from this rule have not yet been analysed. Several studies have discussed temporary social parasitism as an ancestral strategy of social parasitism, a transitional step from normal to parasitic social systems,^17,18^ so this strategy may be a missing link in the evolution of social parasitism. Furthermore, by studying species that deviate from Emery’s rule, it is possible to investigate the evolutionary background of social parasitism from factors other than host relatedness. Therefore, we believe that conducting omics studies on various temporary socially parasitic ant species and their host that deviate from Emery’s rule will help us investigate the universal nature of social parasitism.

An example of this research is the elucidation of the chemosensory system in socially parasitic ants, particularly their interaction with semiochemicals, including chemosensory proteins (CSPs). CSPs are binding proteins that transport lipophilic compounds to olfactory receptors.^19–22^ In ants, CSPs are primarily expressed in chemosensory organs, especially the antennae.^22^ Some CSPs are also known to bind cuticular hydrocarbons—key substances in nestmate recognition—and are believed to play a critical role in this system.^19^ Regardless of whether they follow Emery’s rule, socially parasitic ants must coexist with their host species. This coexistence suggests that socially parasitic ants have a chemosensory system capable of detecting the semiochemicals used by their hosts. Consequently, it is hypothesized that these ants possess and express CSPs similar to those of their hosts. However, the specifics of CSPs in socially parasitic ants remain unclear, likely due to the limited availability of genomic data.

**Fig. 1.**
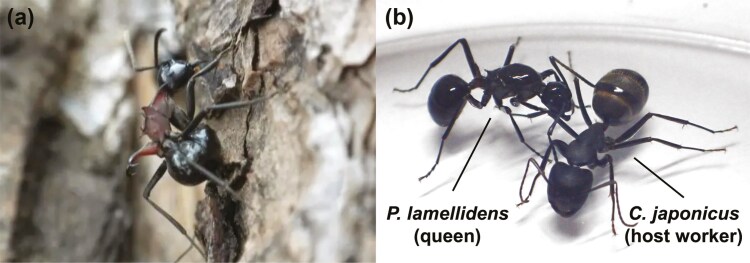
The pictures of ants. a) *P. lamellidens* (worker). b) *P. lamellidens* (queen) and *C. japonicus* (host worker).

*Polyrhachis lamellidens* and its host, *Camponotus japonicus*, can be a useful model to investigate the above question. *P. lamellidens* is a temporary socially parasitic spiny ant belonging to the subfamily Formicinae (Fig. 1). *P. lamellidens* uses several *Camponotus* ant species as hosts, and the newly mated queen takes over the host ant colony in the early stages of social parasitism.^23–33^ The host of *P. lamellidens* is a different genus from them, *Camponotus*, thus it is outside Emery’s rule. In addition, newly mated *P. lamellidens* queen is suggested to recognize its host workers using host semiochemicals, including cuticular hydrocarbons, as cues.^33^ However, the only omics information available for *P. lamellidens* was the transcriptome assembly from our previous study,^32^ and genomic information was lacking. Furthermore, genomic information for the temporary socially parasitic ants and their hosts that deviate from Emery’s rule has not been published.

In this research, we report the chromosomal genome assembly of *P. lamellidens* using the 10× Genomics linked-read and Hi-C sequencing, and the draft genome assembly of its host, *C. japonicus*, with long-read sequencing of the Revio system (PacBio). As a precursor to comparative genomics between *P. lamellidens* and *C. japonicus*, we investigated the diversity and expression patterns of CSPs in both species. These ant genomes provide the essential genetic toolkit for comparative genomic and molecular ecological studies of the evolution of social parasites.

## Materials and methods

### Sampling and rearing

We collected *P. lamellidens* workers and the newly mated queens in Yamanashi, Yamagata, and Niigata Prefecture, Japan (2015 to 2024). *P. lamellidens* workers (single individuals or colonies) were collected from a primeval beech forest and a mixed forest in Nirasaki City, Yamanashi Prefecture, Japan (N 35°43′55″, E 138°29′18″), Oguni-machi, Yamagata Prefecture (N 37°55′13″, E 139°40′54″), and Nagaoka City, Niigata Prefecture, Japan (N 37°25′43.5″, E 138°52′56.8″). The newly mated *P. lamellidens* queens were collected from a mixed forest in Nagaoka City, Niigata Prefecture, Japan (N 37°25′43.5″, E 138°52′56.8″). The newly mated *P. lamellidens* queens and a single worker were reared individually in a plastic tub (4.5 cm long, 2.5 cm wide, 2.0 cm high) which contained moistened tissue under dark (0 L:24 D) conditions at 20 °C. *P. lamellidens* colonies were reared in a plastic tub (20.5 cm long, 10.5 cm wide, 7.0 cm high, or 27.0 cm long, 19.0 cm wide, 5.1 cm in high) with gypsum and a feeding area under dark conditions (0 L:24 D) at 20 °C to 30 °C. The newly mated *P. lamellidens* queens were fed 5 μl of 50% maple syrup every 7 to 10 days. *P. lamellidens* colonies were also fed 1,000 to 5,000 μl of 50% maple syrup, mealworms, and crickets every 7 to 10 days.

We sampled *C. japonicus* larvae (second, third, and fourth stages), a worker, a queen, and males. To establish new colonies, the newly mated *C. japonicus* queens were collected in Tsukuba City, Ibaraki Prefecture, Japan (N 36°06′08″, E 140°13′13″) between 2020 and 2024. *C. japonicus* colonies were reared in a plastic tub (20.0 cm long, 7.0 cm wide, 6.5 cm high, or 9.0 cm diameter, 4.5 cm high) containing the test-tube filled with water, under dark (12 L:12 D) conditions at 20 °C to 30 °C. The colonies were fed 100 to 1,000 μl of 50% maple syrup and crickets every 7 days after the workers emerged. Larvae, a worker, and a queen were sampled from a newly established colony. We also sampled males produced after the queen’s death, when the workers began ovipositing.

### Ant contact testing

The newly mated *P. lamellidens* queen exhibits varying levels of parasitic behaviour (rubbing behaviour) toward host workers, depending on the individual and timing. In this study, we selected individuals with low (slow queen) and high (quick queen) parasitic activity, following the method described by Kurihara et al.^33^

### DNA extraction and genome sequencing

Genomic DNA was extracted from 2 workers of *P. lamellidens* using Genomic-tip 20/G (Qiagen) according to manufacturer’s instructions. Extracted DNA was then used to create linked-read library using Chromium Genome Kit (10× Genomics) according to manufacturer’s instructions, with one modification. After the creation of Gel Bead-In-Emulsions (GEMs) with Chromium Controller (10× Genomics), only 10% of the emulsified solution was used in downstream library preparation to match the smaller target genome size. Prepared Illumina library was then sequenced and basecalled on MiSeq with MiSeq Reagent Kit v3 600 cycles (Illumina). The Hi-C sequencing library of *P. lamellidens* was prepared with EpiTect Hi-C Kit (Qiagen) according to manufacturer’s instructions and was sequenced on NovaSeq X Plus (Illumina) as 150 bp paired-end reads.

Genomic DNA was also extracted from 10 males of *C. japonicus* using the Genomic-tip 20/G (Qiagen) according to the manufacturer’s instructions. The extracted DNA was then used to prepare a library with the SMRTbell Express Template Prep Kit 2.0 (PacBio). Polymerase complexes for the prepared library were generated using the Revio Polymerase Kit (PacBio) and sequenced to 20 Gbp on the Revio (PacBio).

### RNA extraction and cDNA sequencing

Total RNA was extracted from the antennae of *P. lamellidens* (3 workers and 6 newly mated queens) and the whole bodies of *C. japonicus* (1 queen, 1 worker, 1 male, 1 larva, and 1 pupa) for gene expression analysis of CSPs in *P. lamellidens* and to obtain annotation hints for *C. japonicus*. The *P. lamellidens* samples were anesthetized by freezing (4 °C for 2 min and −20 °C for 3 min), after which the antennae were detached from the head. The antennae were washed with 1× PBS and placed in a ZR BashingBead lysis tube (Zymo Research) containing 500 μl of TRIzol Reagent (Life Technologies). These samples were homogenized using a Multi-Beads Shocker at 2,500 rpm for 30 s (Yasui Kikai), and total RNA was extracted with the Direct-zol RNA MicroPrep (Zymo Research) without DNase treatment. For *C. japonicus* samples, whole bodies were placed in 2 ml sample tube (TOMY) containing beads and 200 μl of TRIzol Reagent. The samples were homogenized with a Micro Smash at 3,000 rpm for 30 s (TOMY), after which 600 μl of TRIzol reagent was added. Additionally, 100 μl of 1-bromo-3-chloropropane was added to facilitate RNA extraction, followed by precipitation with 100% isopropanol and 70% ethanol. RNA quantification and quality checks for all samples were carried out using the TapeStation 2200 RNA Screen Tape (Agilent Technologies) or Bioanalyzer High Sensitivity DNA Analysis (Agilent Technologies), Qubit Broad Range or High Sensitivity (BR or HS) RNA assay (Life Technologies), and NanoDrop 2000 (Thermo Fisher Scientific).

Illumina sequence libraries for cDNA sequencing were prepared from 100 to 200 ng of extracted RNA using either the KAPA mRNA Capture Kit and KAPA mRNA HyperPrep Kit (KAPA BIOSYSTEMS) for *P. lamellidens* samples or the NEBNext Ultra II Directional RNA Library Prep with Sample Purification Beads and NEBNext Poly(A) mRNA Magnetic Isolation Module (New England BioLabs) for *C. japonicus* samples. These libraries were prepared according to the manufacturer’s instructions. Amplification of the adapter-ligated cDNA was performed by PCR (16 to 18 cycles). The cDNA libraries were sequenced on the NextSeq 500 or NovaSeq X Plus (Illumina) with 150 bp paired-end or 75 bp single-end reads.

### Genome assembly

In *P. lamellidens*, genome sequence was assembled using Supernova v2.0 software (10× Genomics) with default parameters, outputting in pseudohap mode. In *C. japonicus*, genome sequence (only HiFi reads over 1,000 bp) was assembled using Hifiasm v0.19.5-r587 software^34^ with default parameters. The contaminants contigs in the assembled genome were eliminated by BlobTools v1.1.1^35^ (Supplementary Fig. S1). The assembled genome sequences were subjected to a Diamond BLASTX v2.0.15 search (option: --sensitive, --max-target-seqs 1, --evalue 1e-25)^36^ against the UniProt Knowledgebase (containing Swiss-Prot and TrEMBL) (downloaded on 2024 April). The coverage data were generated and converted by mapping the raw reads to contigs using BWA-MEM v2.2.1^37^ and Samtools v1.17.^38^ The classified contigs as bacterial, plant, fungal, and mitochondrial were removed from the assembled genome. For Hi-C scaffolding in *P. lamellidens*, basecalled and demultiplexed reads totalling 203 Gbp were first filtered using fastp v0.19.4^39^ with -c and -g options, and the filtered reads were processed with HiC Pro v3.1.0.^40^ Qualified reads were used to scaffold the assembly using YAHS v1.2^41^ with default parameters. Scaffolds with length over 10 kbp were retained. Contact map was generated using Juicer 1.6 pipeline.^42^ In *C. japonicus*, since many individuals were used together for genome sequencing, Purge_dups v1.2.5^43^ was employed to identify and remove haplotypes. The completeness of filtered genome assembly was evaluated by BUSCO v5.4.7^44^ with the insecta_odb10 dataset. Snail Plot of filtered genome assembly was visualized by BlobToolKit v4.3.0.^45^ In addition, heterozygosity was estimated by Jellyfish v2.2.10^46,47^ and GenomeScope v2.0^48^ based on K-mer frequency distributions.

Mitochondrial genome of *P. lamellidens* was assembled by NOVOPlasty v4.3.3^49^ from raw sequenced read data. We used the sequence of the mitochondrial gene coding region of cytochrome oxidase subunits I and II (*COI*/*II*) of *P. lamellidens*, sequenced in our previous study,^31^ as the seed input file for the assembly. Annotation and visualization of mitochondrial genome were carried out by GeSeq.^50^

### Genome annotation

Prior to gene annotation, we soft-masked each assembled genome using RepeatModeler v2.0.5 and RepeatMasker v4.1.5.^51^ The soft-masked genomes were used to annotate protein-coding genes using BRAKER v3.0.8.^52^ We used RNA-seq and protein datasets as hints for annotation. RNA-seq data containing queen (NCBI Accession ID: SRX14662390), worker (NCBI Accession ID: SRX14662379), and larvae (NCBI Accession ID: SRX14662378) of *P. lamellidens* as well as queen, worker, male, larva, and pupa of *C. japonicus* (DDBJ Accession No: DRA020043) were trimmed using Trimmomatic v3.0.0 (option: PE -phred33, MINLEN:50)^53^ and then mapped to each soft-masked genome using HISAT v2.2.1.^54^ The resulting mapping files were converted by Samtools v1.17^38^ and were used as annotation hints. The *C. floridanus* protein-coding gene dataset (NCBI Accession ID: GCF_003227725.1, Supplementary Table S1) was also used as annotation hints. From the candidate genes predicted by BRAKER v3.0.8, we extracted only genes that completely or partially overlapped with the sequences given in the hints. In addition, redundant genes were clustered and eliminated by CD-HIT-EST v4.8.1^55,56^ (option: -c 0.97) from the above extracted genes. We determined the sorted genes from the above process to be protein-coding genes and evaluated its competence using BUSCO v5.4.7^44^ with the insecta_odb10 dataset. Functional annotation of the predicted protein-coding genes was performed by the Diamond BLASTX v2.0.15 search (option: --sensitive, --max-target-seqs 1, --evalue 1e-25)^36^ against the UniProt Knowledgebase (downloaded on 2024 April).

### Genome-wide phylogeny and synteny analysis

We used the genome of *P. lamellidens*, *C. japonicus*, and 7 ant species (plus *Apis mellifera* as an outgroup) for genome-wide phylogenetic analysis (Supplementary Table S1). Using the phylogenetic inference pipeline published by Manni et al.^57^ (including AGAT toolkit, BUSCO, MAFFT, trimAl, AMAS, IQ-TREE2), a phylogenetic tree was constructed with a concatenation of single-copy gene sets from BUSCO that are common hits across species. The constructed and aligned concatemers were subjected to phylogenetic analysis based on the maximum likelihood method (model: Q.insect+R5) by IQ-TREE2 with 1,000 ultrafast bootstrap replicates. The phylogenetic tree was visualized by FigTree v1.4.4 (http://tree.bio.ed.ac.uk/software/figtree/).^58^

We analysed the synteny relationship between *P. lamellidens* and *C. japonicus*. Synteny analysis was performed only on the homologous genes found in the phylogenetic analysis and the scaffold in which they are located. BLASTP v2.14.1 (option: e-value: 1E-10)^59^ and MCScanX^60^ were used to calculate the gene synteny and collinearity, and its results were visualized by SynVisio.^61^

### Annotation of CSPs and gene expression analysis

We annotated CSPs from the genome assemblies of *P. lamellidens* and *C. japonicus* using the CSP sequences of *C. japonicus* from a previous study^22^ as a query. Candidate CSPs were identified by performing a BLASTN search (v2.14.1)^59^ against the genome assemblies (e-value: 1E-10) and manually verified using IGV v2.16.2.^62^ The obtained candidate genes and query sequences were further confirmed for their protein domain and motif structures using HMMER v3.1b2^63^ with the Pfam-A database (e-value ≤ 1e-10), DoMosaics v0.95,^64^ and MEME v5.5.7.^65^ Candidate genes that contained the domain (Pfam ID: PF03392) and motif structures similar to those of the query were considered CSPs of *P. lamellidens* and *C. japonicus*. The identified CSPs were aligned and trimmed using MUSCLE v3.8.1551^66^ and Trimal v1.4.rev15.^67^ The alignment data were subjected to phylogenetic analysis using the maximum likelihood method (model: LG + I + G4) in IQ-TREE v2.0.3^68^ with 1,000 ultrafast bootstrap replicates and 1,000 SH-aLRT (Shimodaira–Hasegawa approximate likelihood ratio test) replicates. The phylogenetic tree was visualized using FigTree v1.4.4 (http://tree.bio.ed.ac.uk/software/figtree/).^59^

The gene expression levels of *P. lamellidens* and *C. japonicus* were quantified as transcripts per million (TPM) using Kallisto v0.43.0.^69^ In this analysis, we also utilized the cDNA sequencing data of *C. japonicus* antennae (NCBI Accession ID: DRA002913) obtained from a previous study.^22^ The R package EdgeR v3.18.1^70^ was used to examine the changes in expression between castes of both species, with a false discovery rate (FDR) of <5%, using the glmLRT method.

## Results and discussion

### Genome assembly and annotation

**Fig. 2.**
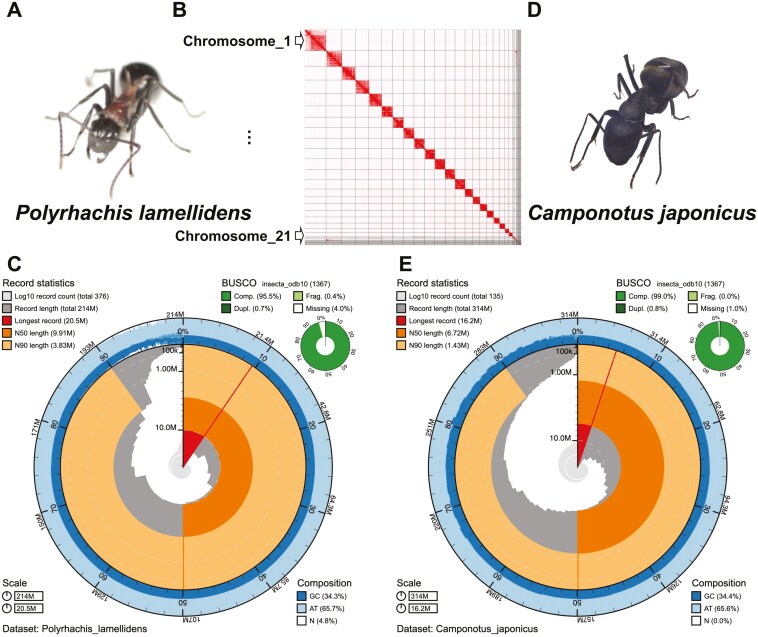
Summary of the *P. lamellidens* and *C. japonicus* genome assemblies. a) *P. lamellidens* (worker). b, c) The contact map and snail plot of the *P. lamellidens* chromosomal genome assembly. The contact map shows the 21 superscaffolds corresponding to chromosomes, ordered from longest to shortest. d) *C. japonicus* (worker). e) The snail plot of the *C. japonicus* draft genome assembly.

**Fig. 3.**
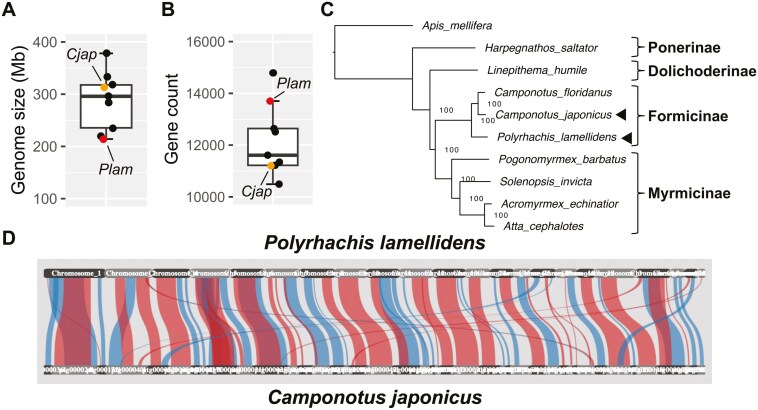
Feature of the *P. lamellidens* and *C. japonicus* genome assemblies. a, b) The genome size and the number of protein-coding genes of 9 ant species. The *Plam* indicates *P. lamellidens*. The *Cjap* indicates *C. japonicus*. c) Phylogenetic relationship among 9 ant species. The ultrafast bootstrap support values are labelled on the branch points of tree. Outgroup is *A. mellifera*. The arrow heads indicate *P. lamellidens* and *C. japonicus*. d) Synteny relationship between *P. lamellidens* and *C. japonicus*. Alignment orientation is showed as regular (blue) or reversed (red). The synteny share of collinear genes is 84.5%.

The chromosomal genome of *P. lamellidens* is 214.1 Mb in size and comprises 376 scaffolds (Fig. 2C). The contact map identified 21 superscaffolds (Fig. 2B), which correspond to the number of chromosomes previously reported in the genus *Polyrhachis*.^71^ The draft genome of *C. japonicus* is 314.2 Mb in size and comprises 135 scaffolds (Fig. 2E). The genome size of *P. lamellidens* and *C. japonicus* falls within the general range of ant genome size (Fig. 3A). The heterozygosity of the *P. lamellidens* genome is 0.413%, and that of the *C. japonicus* genome is 0.106%, as estimated from the raw-read-based k-mer analyses (Supplementary Fig. S2). In *P. lamellidens*, the median scaffold length is 9.91 Mb (N50) and the longest scaffold is 20.5 Mb (Fig. 2C). In *C. japonicus*, the median contig length is 6.72 Mb (N50) and the longest contig is 16.2 Mb (Fig. 2E). BUSCO (dataset: insecta_odb10) evaluated the assembly completeness of 95.5% in *P. lamellidens* (Fig. 2C), and 99.0% in *C. japonicus* (Fig. 2E).

We confirmed that 11.71% of the genome consists of repeat elements in *P. lamellidens*, and 36.13% in *C. japonicus* (Supplementary Table S2). Most of the annotated repeat elements were unclassified in *P. lamellidens* (6.27%) and *C. japonicus* (20.44%). In *P. lamellidens*, among the classified repeat elements, DNA elements represent 1.00%, long terminal repeat (LTR) elements represent 0.32%, long interspersed nuclear elements (LINEs) represent 0.31%, and short interspersed nuclear elements (SINEs) represent 0.02%. In *C. japonicus*, among the classified repeat elements, DNA elements represent 5.43%, LTR elements represent 3.51%, LINEs represent 2.94%, and SINEs represent 0.12%. In the previous studies, a similar level (15.10% to 34.16%) of repeat elements was observed in the genomes of ant species belonging to the same subfamily Formicinae as *P. lamellidens* and *C. japonicus*.^72–76^

We predicted 13,703 protein-coding genes and 17,251 transcripts from the chromosomal genome of *P. lamellidens*. We also predicted 11,207 protein-coding genes and 14,507 transcripts from the draft genome of *C. japonicus*. A total of 9,212 genes of *P. lamellidens* (82.1%) and 9,521 genes of *C. japonicus* (84.9%) were functionally annotated against the UniProt Knowledgebase. The number of predicted genes of *P. lamellidens* and *C. japonicus* are comparable to the other 7 ant species, falling within the range between the minimum and maximum values of the box plot (Fig. 3B). The protein-coding genes show 95.0% BUSCO (dataset: insecta_odb10) completeness for *P. lamellidens* and 98.1% for *C. japonicus* (Supplementary Table S3).

The mitochondrial genome assembly of *P. lamellidens* is 1 circular contig of 16.3 kb size. This genome contains 13 protein-coding genes, 2 ribosomal RNAs, and 12 transfer RNAs (Supplementary Fig. S3) and resembles the genome size (15.8 to 18.9 kb) of Formicinae ant mitochondria reported in previous studies.^77–82^

From the above results, we believe that the assembly and annotation of a nuclear and mitochondrial genome of sufficient quality is achieved.

### Genome-wide phylogenetic analysis among several ant species

We constructed a phylogenetic tree using 1,132 BUSCO genes that are common hits across 9 ant species and *A. mellifera* (Fig. 3C). Our phylogenetic tree showed similar results to those of previous studies using some genetic regions.^83,84^ Several studies have suggested that the genus *Polyrhachis* is relatively closely related to *Camponotus*,^78,83,84^ and our phylogenetic tree also supports this theory by placing *P. lamellidens* and *C. japonicus* in the same clade as *C. floridanus*. We provide the first genome-wide support for phylogenetic relationships among *P. lamellidens* and *C. japonicus*.

### Synteny analysis between *P. lamellidens* and *C. japonicus*

Synteny analysis between *P. lamellidens* and *C. japonicus* was performed for 1,132 BUSCO genes in the 9 ant species (plus *A. mellifera*) used in the previous phylogeny analysis (Fig. 3D). Out of the 36 scaffolds identified in *P. lamellidens* (where 1,132 BUSCO genes are located), 22 scaffolds were mapped to the 42 scaffolds in *C. japonicus*. Since the synteny of collinear genes is 84.5%, and most of the scaffolds of *P. lamellidens* correspond to those of *C. japonicus*, the genomes of the 2 ant species show a high level of synteny. These results support the validity of the *P. lamellidens* and *C. japonicus* genome sequenced in this study.

### The clustering and expression patterns of CSPs of *P. lamellidens* and *C. japonicus*

**Fig. 4.**
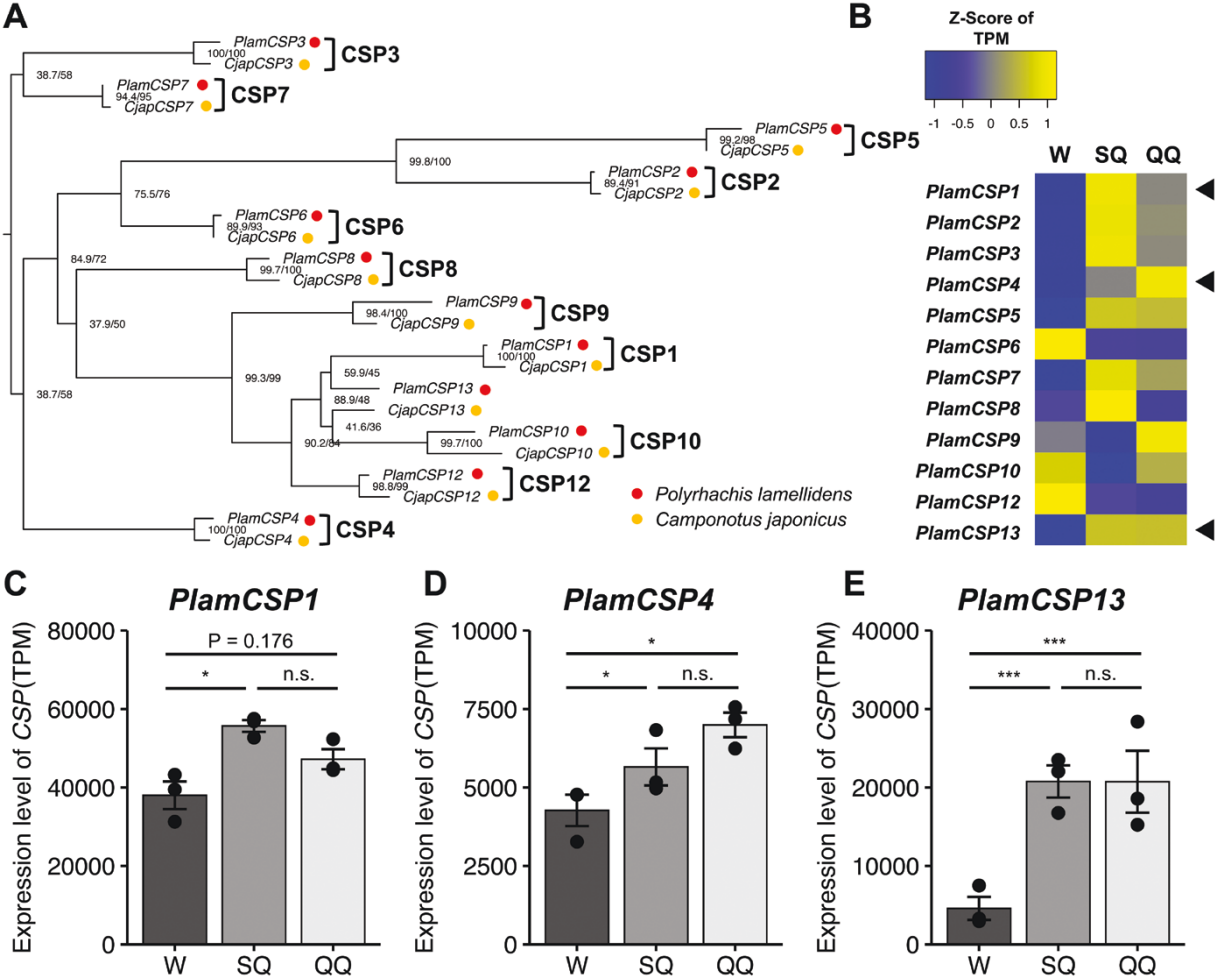
Summary of CSPs in *P. lamellidens* and *C. japonicus*. a) Phylogenetic relationship of CSPs in *C. japonicus* and *P. lamellidens*. */* is SH-aLRT support/ultrafast bootstrap support. b) The heatmap of CSPs expression in *P. lamellidens*. TPM was converted to a *Z* score. The arrow heads indicate DEGs. c) The expression levels of CSPs detected as DEG in *P. lamellidens*. *Significant difference: FDR < 0.05; ***significant difference: FDR < 0.001; n.s., non-significant difference, *n* = 3. Error bar: SE. W: worker; SQ: slow queen; QQ: quick queen.

Manual annotation using the *C. japonicus* CSP sequences identified in Hojo et al.^22^ allowed us to identify 12 CSPs in both the *C. japonicus* and *P. lamellidens* genome assemblies obtained in this study. The number of CSPs in ants generally ranges from 6 to 21,^20–22^ and the CSPs identified in this study fell within that range. For *C. japonicus*, the sequences obtained were identical to those identified in Hojo et al.,^22^ and no novel sequences were discovered. Phylogenetic analysis revealed that the CSPs of *P. lamellidens* and *C. japonicus* are generally one-to-one orthologs, with the exception of *CjapCSP13* and *PlamCSP13* (Fig. 4A). This analysis indicates that *P. lamellidens* has a similar CSP profile despite being in a different genus from its host *C. japonicus* (deviating from Emery’s rule). This suggests that *P. lamellidens* may possess a chemosensory system adapted to the chemical communication system of *C. japonicus*.

The gene expression analysis of the antennae in *P. lamellidens* and *C. japonicus* revealed that several CSPs were identified as differentially expressed genes (DEGs) (Fig. 4B–E, Supplementary Fig. S4). For *C. japonicus* CSPs, we largely replicated the results from Hojo et al.,^22^ showing a pattern of differential expression depending on sex (Supplementary Fig. S4). In *P. lamellidens*, many CSPs were more highly expressed in newly mated queens compared to workers (Fig. 4B), with *PlamCSP1*, *4*, and *13* being significantly more highly expressed in newly mated queens (except between workers and quick queens for *PlamCSP1*) (Fig. 4C–E). Although the newly mated *P. lamellidens* queen does not engage in typical social behaviours such as foraging and brood care like workers, we hypothesize that she expresses CSPs at levels equal to or higher than those of workers to facilitate host recognition by actively sensing the host’s cuticular hydrocarbons. Indeed, Kurihara et al.^33^ demonstrated that the newly mated *P. lamellidens* queen performs parasitic behaviours (such as rubbing behaviour) by recognizing cuticular compounds, including cuticular hydrocarbons, from host ants. Furthermore, *CjapCSP1*, the orthologous gene of *PlamCSP1*, is known to bind to a wide range of cuticular hydrocarbon components without disrupting their profile.^19^ Since *PlamCSP1* is the ortholog of *CjapCSP1*, it is presumed that this gene also enables binding to cuticular hydrocarbons. The same function may be present in *PlamCSP13*, which is duplicated from the *PlamCSP1* lineage. Based on these findings, we hypothesized that the newly mated *P. lamellidens* queen expresses high levels of CSPs, particularly *PlamCSP1* and *PlamCSP13*, to find host workers and perform parasitic behaviours.

No significant difference in CSPs expression was observed between slow and quick newly mated queens (Fig. 4C–E). This suggests that CSPs in newly mated queens are unlikely to be directly associated with the activity level of parasitic behaviours towards the host. We hypothesize that the activity of parasitic behaviours is more likely related to the olfactory pathway downstream of CSP binding to host cuticular compounds.

We believe that our analyses provide an example of the commonalities between socially parasitic ants and their hosts that deviate from Emery’s rule, at least in terms of peripheral chemosensory systems such as CSPs. This finding could be a crucial aspect of the social parasitism strategy in any socially parasitic ants. Since our study focused on only one pair of socially parasitic ants and their hosts that deviate from Emery’s rule, it is important to verify whether these findings are universally applicable to other socially parasitic ants and their hosts with similar characteristics. In addition to *P. lamellidens*, there are 2 other socially parasitic ants in the genus *Polyrhachis* that also deviate from Emery’s rule,^85,86^ and these species could serve as valuable models for testing the hypotheses discussed above.

## Conclusion

This study presents the chromosomal and draft genome assemblies of the temporary socially parasitic spiny ant *P. lamellidens* and its host *C. japonicus*, with genome-wide phylogenetic analysis confirming the position of both species. Additionally, we were able to infer part of the role of CSPs in the social parasitism strategy of *P. lamellidens*. We believe that this study provides the molecular biological and bioinformatic basis for studying the strategy of social parasitism in *P. lamellidens.* The genome assemblies of *P. lamellidens* and its host species will help to verify the universal mechanisms underlying the evolution of the social parasitism strategy.

## Supplementary Material

dsaf005_suppl_Supplementary_Materials

## Data Availability

The *Polyrhachis lamellidens* nuclear and mitochondrial genome assemblies are available at DDBJ Accession No.: AP038989-AP039364 and AP036026. The raw sequence data of *P. lamellidens* genome are available at DDBJ Accession No.: DRA019757 and DRA019757. The *Camponotus japonicus* nuclear genome assembly is available at DDBJ Accession No.: BAAGFG010000001–BAAGFG010000135. The raw sequence data of *C. japonicus* genome are available at DDBJ Accession No.: DRA019707. Raw sequence reads of *P. lamellidens* and *C. japonicus* cDNA were available at DDBJ Accession No.: DRA020042 and DRA020043.
