# Supplementary material for: Genome assembly of the temporary socially parasitic spiny ant Polyrhachis lamellidens and its host Camponotus japonicus
Source: DNA Res. 2025 Mar 1;32(2):dsaf005. doi: 10.1093/dnares/dsaf005 (PMC12016558; doi:10.1093/dnares/dsaf005)
Supplement: dsaf005_suppl_Supplementary_Materials [file dsaf005_suppl_supplementary_materials.docx]

**Supplementary Fig. 1.** **Results of the BlobTools analysis.** ReadCovPlot of the *P. lamellidens* (A) and *C. japonicus* (C) draft genome assembly before removal of contaminated contigs. BlobPlot of the *P. lamellidens* (B) and *C. japonicus* (D) draft genome assembly before removal of contaminated contigs. In the BlobPlot, the plots show the taxonomic assignment at the phylum rank level. These plots are distributed according to GC% and coverage. The diameter of the plots is scaled proportionally to the sequence length.

**Supplementary Fig. 2.** **Results of the GenomeScope analysis.** GenomeScope profiles for *P. lamellidens* (A) and *C. japonicus* (B). len: expected total genome length, uniq: percent unique sequence of the genome, het: heterozygosity, kcov: k-mer coverage for heterozygous bases, err: error rate of the reads, dup: average rate of read duplications, k: k-mer size, observed: the observed k-mer profile, full model: the estimated model, unique sequence: unique sequences, errors: error sequences, k-mer peaks: peak of the k-mer coverage distribution.

**Supplementary Fig. 3.** **Overview of the mitochondrial genome assembly of *P. lamellidens*.**


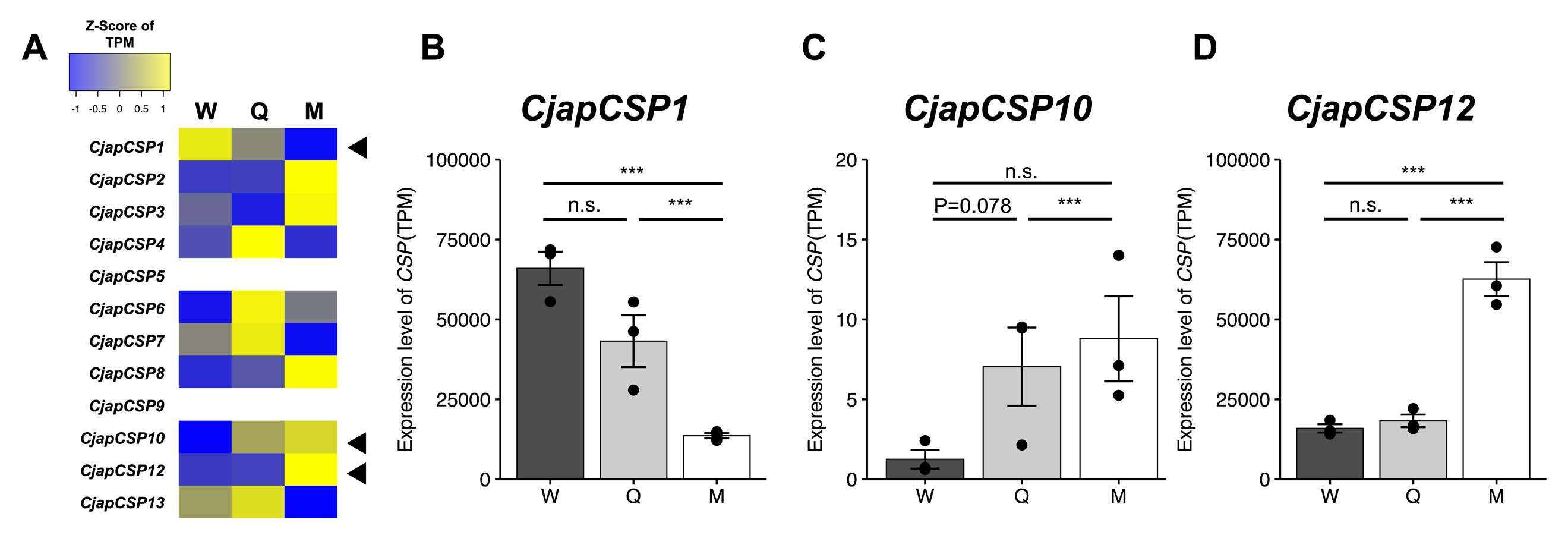


**Supplementary Fig. 4.** **Expression patterns of chemosensory proteins (CSPs) in *C. japonicus*.** (A) The heatmap of CSPs expression in *C. japonicus*. TPM was converted to a Z score. The arrow heads indicate differentially expressed genes (DEGs). (B-D) The expression levels of CSPs detected as DEG in *C. japonicus*. ***, significant difference: FDR < 0.001; n.s., non-significant difference, n = 3. Error bar: SE. W: worker, Q: alate queen, M: male.

**Supplementary Table S1**. The NCBI accession IDs of the species targeted in this study.

| Species | Subfamily (ant only) | NCBI Accession ID |
| --- | --- | --- |
| *Apis mellifera* | - | GCF_003254395.2 |
| *Harpegnathos saltator* | *Ponerinae* | GCF_003227715.2 |
| *Linepithema humile* | *Dolichoderinae* | GCF_000217595.1 |
| *Camponotus floridanus* | *Fromicinae* | GCF_003227725.1 |
| *Pogonomyrmex barbatus* | *Myrmicinae* | GCF_000187915.1 |
| *Solenopsis invicta* | *Myrmicinae* | GCF_016802725.1 |
| *Atta cephalotes* | *Myrmicinae* | GCF_000143395.1 |
| *Acromyrmex echinatior* | *Myrmicinae* | GCF_000204515.1 |

**Supplementary Table S2**. Results of RepeatModeler and RepeatMasker analysis. The table shows the percentage of different repeat elements and small RNAs identified in *P. lamellidens*.

|  | Number of elements | | Length occupied (bp) | | Percentage of sequence (%) | |
| --- | --- | --- | --- | --- | --- | --- |
|  | ***P. lamellidens*** | ***C. japonicus*** | ***P. lamellidens*** | ***C. japonicus*** | ***P. lamellidens*** | ***C. japonicus*** |
| Short interspersed nuclear elements (SINEs) | 183 | 1,662 | 34,404 | 390576 | 0.02 | 0.12 |
| Long interspersed nuclear elements (LINEs) | 1,540 | 12,371 | 6,57,019 | 9,240,791 | 0.31 | 2.94 |
| Long terminal repeat (LTR) elements | 829 | 9,879 | 6,77,006 | 11,044,631 | 0.32 | 3.51 |
| DNA elements | 6,176 | 30,941 | 2,138,327 | 17,075,346 | 1.00 | 5.43 |
| Unclassified | 59,755 | 170,284 | 13,427,462 | 64,219,042 | 6.27 | 20.44 |
| Small RNA | 183 | 1,739 | 34,404 | 440,402 | 0.02 | 0.14 |
| Satellites | 0 | 891 | 0 | 331267 | 0.00 | 0.11 |
| Simple repeats | 144,526 | 166,521 | 6,898,066 | 8,834,496 | 3.22 | 2.81 |
| Low complexity | 22,626 | 27310 | 1,197,870 | 1488428 | 0.56 | 0.47 |

**Supplementary Table S3**. Results of BUSCO analysis (dataset: insecta_odb10) of predicted protein-coding genes.

|  | BUSCO scores (%) | |
| --- | --- | --- |
|  | ***P. lamellidens*** | ***C. japonicus*** |
| Complete BUSCOs | 95.0 | 98.1 |
| Complete and single-copy BUSCOs | 94.6 | 97.4 |
| Complete and duplicated BUSCOs | 0.4 | 0.7 |
| Fragmented BUSCOs | 0.7 | 0.3 |
| Missing BUSCOs | 4.3 | 1.6 |
